# Genome Sequence of Torovirus Identified from a Pig with Porcine Epidemic Diarrhea Virus from the United States

**DOI:** 10.1128/genomeA.01291-14

**Published:** 2014-12-18

**Authors:** Srivishnupriya Anbalagan, Jessica Peterson, Brent Wassman, Josh Elston, Karen Schwartz

**Affiliations:** Newport Laboratories, Inc., Worthington, Minnesota, USA

## Abstract

Porcine torovirus (PToV) strain PToV-NPL/2013 was identified from a pig that tested positive for porcine epidemic diarrhea virus (PEDV). The spike protein-encoding gene from PToV-NPL/2013 had 92% identity with PToV-SH1, suggesting that PToV circulating in the United States is slightly different from the isolates circulating in China. To our knowledge, this is the first report of PToV in the United States.

## GENOME ANNOUNCEMENT

Toroviruses (ToV) are considered emerging viruses with a possibility of zoonotic transmission (1). They are enveloped positive-stranded RNA viruses and belong to the family *Coronaviridae*, order *Niodvirales* (1, 2). ToV infections are generally asymptomatic, but occasionally they can cause severe diarrhea when concurrent infections occur with other enteric pathogens (1, 3–5). Porcine ToVs (PToV) are identified in piglets worldwide and are prevalent in Africa, Europe, and Asia (6–10). Because there is no *in vitro* system to grow PToV, there is very little information known about the virus. To date, there is only one whole-genome sequence of PToV available in GenBank.

In January 2014, porcine fecal/vomit swabs were submitted to Newport Laboratories for diagnostic testing. Samples were tested for common enteric viruses (rotaviruses A, B, and C; porcine enterovirus 8 and 9; porcine teschovirus; and porcine epidemic diarrhea virus [PEDV]). Samples were positive for group C rotavirus (cycle threshold [*C_T_*], 31.18), PEDV (*C_T_*, 28.47), porcine enterovirus 9, and porcine teschovirus. To further characterize RNA viruses in the sample, we conducted metagenomics using an Ion Torrent Personal Genome Machine with the methodology previously described (11). Sequence assembly was conducted *de novo* using the DNAStar Lasergene 11 Core Suite. BLAST analysis of the assembled contigs identified PEDV, porcine enterovirus 9, porcine teschovirus, and PToV. Since we were interested in PToV, we filled any gaps in the PToV sequence by Sanger sequencing. To our knowledge, this is the first report of PToV identified in the United States.

The complete genome contained 28,305 nucleotides (nt) and was 92% identical to that of porcine torovirus SH1 (PToV-SH1) (NC_022787). The 5′ untranslated region (UTR) and 3′ UTR were 792 and 196 nucleotides in length, respectively. The polyprotein-encoding open reading frame (ORF1ab) was identified from nucleotides 794 to 20910 and encoded a polyprotein of 6,706 amino acids. ORF1a was identified from 794 to 14,047 and encoded a replicase of 4,418 amino acids. Genes encoding spike (S), membrane (M), hemagglutinin-esterase (HE), and nucleocapsid (N) proteins were 4,722, 702, 1,284, and 492 nt in length, respectively, and had predicted proteins with 94, 99, 92, and 96% identity to PToV-SH1, respectively. The spike protein-encoding gene from PToV-NPL/2013 showed only 92% identity to that of PToV-SH1, suggesting that the S gene of PToV circulating in the United States is slightly different from the isolates circulating in China. Since spike is a major glycoprotein present on the viral envelope and is shown to bind host cell receptors, induce virus-mediated cell fusion, and elicit neutralizing antibodies upon infection (12, 13), differences in the PToV-NPL/2013 sequence might alter viral-host interaction. The HE gene from PToV-NPL/2013 had 80 to 95% identity to other PToV isolates in GenBank, suggesting that HE is more diverse among PToV isolates. Genes encoding N and M proteins were 95% and 93 to 97% identical to other PToV isolates in GenBank. Although we identified PToV along with other viruses in a PEDV-positive diarrheal sample, further work is necessary to determine the role of PToV in PEDV-associated diarrhea.

### Nucleotide sequence accession number.

The complete genome sequence of PToV-NPL/2013 has been deposited in GenBank under the accession number KM403390.
